# Extra-Intestinal Manifestation of Celiac Disease in Children

**DOI:** 10.3390/nu10060755

**Published:** 2018-06-12

**Authors:** Hilary Jericho, Stefano Guandalini

**Affiliations:** Department of Pediatrics, Section of Gastroenterology, Hepatology and Nutrition, University of Chicago Celiac Disease Center-Comer Children’s Hospital, Chicago, IL 60637, USA; guandalini@peds.bsd.uchicago.edu

**Keywords:** extra-intestinal, gastrointestinal, celiac disease

## Abstract

The aim of this literature review is to discuss the extra-intestinal manifestations of celiac disease within the pediatric celiac population.

## 1. Overview

Celiac disease (CD) is a complex autoimmune disease that is triggered by the ingestion of gluten (the major storage protein in wheat, rye, and barley) in genetically predisposed individuals, leading to elevated titers of celiac-specific autoantibodies and resulting in a variable degree of small intestinal inflammation and a wide range of gastrointestinal and extra-intestinal manifestations. 

The extra-intestinal manifestations of CD seen most often in the pediatric population include, but are not limited to, short stature, delayed puberty, dental enamel hypoplasia, osteopenia/osteoporosis, iron-deficiency anemia refractory to oral iron supplementation, recurrent stomatitis, liver and biliary disease, dermatitis herpetaformis, arthralgia/arthritis, headaches, ataxia, peripheral neuropathy, epilepsy, behavioral changes, and psychiatric disorders and alopecia. 

While the prevalence of extra-intestinal manifestations of CD is similar between the pediatric and adult populations (60% and 62%, respectively), specific extra-intestinal manifestations and rates of improvement differ. While short stature is the most common extra-intestinal manifestation of CD in children, iron deficiency anemia is most common in adults. The other more commonly encountered extra-intestinal manifestations in both children and adults include fatigue and headaches. Additionally, on average, children appear to have much greater and faster rates of improvement as compared to adults [1,2]. 

It has been shown that children with extra-intestinal manifestations of CD as the main presenting symptom have a more severe degree of villous atrophy than those that are presenting with gastrointestinal manifestations or asymptomatic patients that were detected through screening [2]. The exact etiology for this finding is uncertain. It is not then surprising, though, that at 24 months after starting a strict gluten free diet (GFD), both children and adults with CD show greater and faster rates of improvements in gastrointestinal (90% and 86%, respectively) versus extra-intestinal manifestations of CD (87% and 80%, respectively), which is possibly owing to the more severe histologic findings and more complex mechanism involved with extra-intestinal manifestations. Overall children show greater rates of extra-intestinal symptom resolution as compared to adults and males show greater rates of improvement as compared to females. Factors that appear to predict better rates of symptom resolution after the initiation of a strict GFD include a strong family history of CD, shorter durations of symptoms prior to the diagnosis of CD (those with longer duration of symptoms have greater risk of an altered gut-brain axis setting off a cycle of amplified pain [3]), and strict adherence to the GFD [4].

## 2. Short Stature and Delayed Puberty

Short stature is the most commonly encountered extra-intestinal manifestation of CD in children, being found in roughly one-third of all new pediatric celiac diagnoses. While it can be directly related to malabsorption of nutrients, it should completely reverse once a child is strictly adherent to a GFD. In fact, within 24 months of starting a strict GFD, celiac children should attain appropriate catch up growth and return to their expected trajectory for height. However, if a child is diagnosed post-puberty, their chances for catch up growth are much decreased as the child has likely missed their window. Thus, for post-pubertal patients with short stature, a bone age determination is important to best predict the child’s capacity for additional height growth [1]. If short stature in a prepuberal patient persists beyond 24 months on a strict GFD, it is imperative that the physician start an additional investigation for other missed comorbidities. 

In the 2017 study by Jericho et al. [1], 28% of children with persistent short stature despite strict adherence to the GFD had another missed comorbidity (inflammatory bowel disease, food aversion, Turner Syndrome, or growth hormone deficiency) requiring alternate treatments. Therefore, one must never continue to attribute ongoing short stature to CD, once it appears that the CD has been adequately treated [1]. 

Delayed puberty is another common manifestation of CD affecting roughly 10% of new pediatric celiac patients [1]. Delayed puberty is defined by a lack of physical or hormonal signs of puberty at the age of usual onset. Visible secondary sexual development usually begins when girls achieve a bone age of 11 years and boys achieve a bone age of 12 years. In girls, a lack of breast development by 13 years, or a lack of menarche within three years after breast development or by 16 years is considered to be abnormal. For boys, no testicular enlargement by 14 years or a delay in development for five years or more after onset of genitalia enlargement is considered abnormal. In the case of CD, this delay in puberty is directly related to malabsorption and malnutrition, and should resolve on a GFD, which should prevent any long-term complications and restore normal maturation. If the delay in puberty fails to improve within 12–24 months of starting a GFD, it is imperative that the patient be referred to endocrinology for further evaluation of other underlying defects within the reproductive system [5]. 

## 3. Dental Enamel Hypoplasia and Recurrent Stomatitis

Dental Enamel Hypoplasia can be seen in as many as 40–50% of new pediatric celiac patients as compared to 6% of the healthy population and it leads to the appearance of white, yellow, or brown spots on the teeth with a mottled or translucent-looking appearance [6]. While there is no consensus as to the exact mechanism by which CD leads to dental enamel hypoplasia it is hypothesized that it is secondary to malabsorption of calcium in addition to genetic and immunological factors disturbing the normal process of amelogenesis [7]. Dental enamel hypoplasia occurring in deciduous teeth, within a symmetrical and chronological manner, and detectable in all quadrants of the dentition is most suggestive of an underlying chronic malabsorptive disease, such as CD, as opposed to non-symmetric defects, which are considered to be non-specific [8]. When dental enamel hypoplasia occurs in a child’s permanent teeth, this is most often permanent and will not improve after adopting a strict GFD [9]. 

Roughly 46% of children with CD also suffer from aphthous stomatitis as compared to 20% of the healthy population. It is not yet established whether aphthous ulcers are a direct manifestation of CD or if they occur due to the indirect effects of malabsorption [10]. It is speculated that systemic CD leads to an imbalance in the oral ecosystem that impairs oral health conditions, leading to both dental enamel hypoplasia and aphthous stomatitis. The impact of oral ecosystem alterations, including a change to salivary flow rate and alterations of the hard and soft oral tissues, and its impact on the development of dental enamel defects and aphthous ulcers are being closely evaluated. In a study by Mida et al. in 2011, there did not appear to be significant differences in salivary factors between celiac patients and healthy controls [11], but there were significant differences in the quantity of leukocytes that are present in patients with infectious processes and/or other systemic diseases involving the immune system as compared to healthy controls. They showed that 80% of celiac children who complied with a strict GFD had the complete resolution of leukocyte presence in oral smears as compared to patients with poor adherence to the diet. These non-adherent patients continued to show elevated leukocyte levels and it was hypothesized that these leukocytes in oral smears are what contribute to the formation of the recurrent aphthous ulcers [12] when the appropriate genetic predisposition is present [13]. Others speculate that the direct presence of gluten within the oral cavity may stimulate lymphocyte activity directly into the oral mucosa [11]. Aphthous stomatitis in celiac patients show high rates of resolution on a strict GFD [1]. 

## 4. Osteopenia/Osteoporosis

Osteopenia (reduced bone density) and osteoporosis (reduced bone density leading to weak and brittle bones) are the most frequent bone related complications of CD, and can ultimately lead to bone fragility and a high prevalence of bone fractures if CD is left untreated. Roughly 75% of pediatric celiac patients at the time of diagnosis will have osteopenia, while only 30% have osteoporosis. 

Osteoblasts, which are derived from mesenchymal stem cells, are responsible for new bone formation, while osteoclasts, which are differentiated monocyte-derived cells, are involved in bone matrix removal. Through complicated mechanisms, bones are constantly remodeled through the process of resorption and formation, and in a balanced manner, to limit ultimate bone loss. Nutrition plays a very important role in this bone homeostasis, with the main players being vitamin D, calcium, and minerals, which are predominantly absorbed in the proximal small bowel [14]. While our bodies strive to produce strong bones, the ultimate goal of the human body is to maintain adequate serum calcium levels. When calcium levels are adequate the body favors bone formation. Reduced serum calcium levels, though, leads to parathyroid gland stimulation, increased production of parathyroid hormone (PTH), subsequent bone resorption, and the release of stored calcium to bring the serum calcium levels back into equilibrium. 

Untreated CD to leads to inflammation and villous atrophy in the proximal small bowel, malabsorption of calcium, and low serum levels. This, in turn, leads to secondary hyperparathyroidism, the release of calcium and phosphate from the bone matrix, and the thinning of bones [15]. There is also now evidence to suggest that, in addition to low calcium levels leading to thinning of bones, the chronic release of proinflammatory cytokines, hormonal components, and other misbalanced bone remodeling factors can also predispose celiac patients, on or off the GFD, to mineral metabolism derangements. Specifically, cytokines interleukin 1 beta (IL-1β), interleukin 6 (IL-6), tumor necrosis factor alpha (TNF-α), and interferon gamma (IFN-γ) has been implicated in bone loss during CD [16,17,18,19]. Lastly, there have also been recent advances in the identification of receptor activators of nuclear factor kappa B/receptor activator of nuclear factor kappa B-ligand (RANK/RANKL) signaling system, in addition to the discovery of osteoprotegerin (OPG), a protein that may protect from excessive bone reabsorption. Bone homeostasis is maintained through the balance of the reabsorbing activity of RANKL and the decoy receptor OPG. It has been shown that the OPG/RANKL ratio is significantly lower in celiac patients with recovery of intestinal mucosa as compared to healthy controls that are positively correlating with their lower bone mass density [20]. 

Strict adherence to the GFD is the first-choice therapy for the treatment of osteopenia and osteoporosis in children as it leads to healing of the small bowel and the reversal of intestinal malabsorption. By 12 months on a strict and balanced GFD, most children will have near complete re-mineralization of the bones, even without additional vitamin D and calcium supplementation [21,22]. A greater improvement at 12 months is seen in pediatric celiac patients with GI symptoms as compared to non-GI symptoms, which is likely explained by a delay in the diagnosis of patients lacking GI symptoms leading to more extensive disturbances in bone metabolism at the time of celiac diagnosis [23]. 

## 5. Iron-Deficiency Anemia

While iron-deficiency anemia is one of the most common extra-intestinal manifestations at diagnosis in adult CD patients (50%), and it is only found in roughly 10–15% of new pediatric celiac patients [1,2,24]. This discrepancy can be attributed to delayed diagnoses of CD in adults given the propensity for less typical gastrointestinal celiac symptoms than that seen in children. Iron is predominantly absorbed in the first portion of the small bowel, the duodenum, which is the main portion of the bowel affected by CD. CD induced duodenal inflammation subsequently leads to the malabsorption of iron and resultant iron-deficiency anemia. This anemia will often temporarily improve with iron supplementation only to recur when discontinued given the ongoing iron malabsorption from the untreated CD. Eighty-four percent of celiac children receiving iron supplementation and with strict adherence to a GFD had the complete recovery of their iron stores by 12–24 months [1].

## 6. Liver and Biliary Disease

Liver disease is seen in up to 50% of new pediatric celiac patients [25]. While is it possible for celiac patients to have more severe liver disease, such as autoimmune hepatitis, primary biliary cirrhosis, and sclerosing cholangitis, the majority develop a benign hypertransaminasemia or “celiac hepatitis”. While it is not clear why damage to the liver occurs in CD, it is felt to be likely secondary to damaged gut mucosa with resulting increased gut permeability allowing for endotoxins from gut bacteria to reach the portal vein. Once in the liver, these endotoxins trigger a toll-like receptor-mediated inflammatory response from immune cells, ultimately leading to inflammation and liver damage [26]. Patients with “celiac hepatitis” have excellent response rates to a strict GFD, with a 75–95% rate of complete liver enzyme normalization by 12–24 months [1,27].

## 7. Dermatitis Herpetiformis

Dermatitis Herpetiformis (DH) is rather rare in pediatric celiac patients with rates of roughly 5% or less. DH is a bilateral, itching, blistering skin disease that typically presents as a rash on the elbows, knees, and buttocks. The diagnosis is confirmed by a skin biopsy with direct immunofluorescence demonstrating granular immunoglobulin A (IgA) deposits in the papillary dermis. The majority (but not all) of these patients will also have celiac specific enteropathy in the small intestine at diagnosis as well. The rash responds well to a strict GFD with near 100% rates or resolution in children [1,28]. While some may receive additional medical therapy with diamino-diphenyl sulfone (dapsone) at diagnosis, most celiac patients can be successfully weaned off the medication with time and remain well controlled on a gluten free diet alone [28]. 

## 8. Arthralgia/Arthritis

Musculoskeletal manifestations, including arthralgia and arthritis, are seen in roughly 5–10% of new celiac pediatric patients [1]. In a paper by Garg et al. in 2017, the authors investigated the prevalence of early joint involvement in children with CD through the use of musculoskeletal ultrasound, which is felt to be superior to conventional radiology in detecting a wide array of early inflammatory and structural abnormalities in joints. They compared children aged 2–18 with newly diagnosed CD on a strict GFD as compared to children with CD who had been on a GFD for at least six months. Ultrasonographic assessment showed the presence of at least one abnormality in the joints of 32% of newly diagnosed celiac patients as compared to only 3% of those on the diet for at least six months. The most frequently involved joint was the knee with findings, including joint effusion, synovial hypertrophy, and joint effusion with synovitis. Other joints, less often affected, included the hip and ankle. Interestingly, the majority of patients with ultrasonographic evidence of joint abnormalities were asymptomatic, suggesting a subclinical synovitis. The lower rates of joint involvement found in children on a GFD for more than six months is suggestive that the GFD may lead to improvements in the joint abnormalities associated with CD [29]. Limitations of this study included the small sample size and lack of healthy controls for comparison, though. To better understand the rates of subclinical synovitis in the healthy population, Breton et al. in 2011 assessed 41 healthy control pediatric patients, none of whom showed signs of subclinical synovitis evaluated by musculoskeletal ultrasonography [30]. The children studied, however, were French with a mean age of nine years old as compared to Indian children with a mean age of 6 in Garg, et al.’s study, thus making this healthy control group not well suited for such a comparison.

## 9. Headaches, Peripheral Neuropathy, Ataxia and Epilepsy

While headaches can be seen in up to 20–30% of children with new CD, rates of peripheral neuropathy and epilepsy are rarer. While these diseases may occur simultaneously by chance, there are several reports demonstrating neurological improvement following the introduction of a strict gluten free diet supporting a causative association. 

Headaches are the most common neurological symptoms seen in pediatric CD. The exact mechanism by which CD leads to headaches in is unclear, but it is speculated that it may be secondary to a lack of vitamins, macro elements, such as magnesium [31], low levels of serotonin [32], which are the direct result of the celiac associated malabsorption. An alternate hypothesis is that the impaired immune response results in an imbalance of pro-inflammatory cytokines in response to ingested gluten, leading to altered vascular tone, and subsequently, the onset of the headache [33]. Most pediatric patients show excellent rates of headache improvement on a strict GFD, approaching 100% [1,34,35].

The most common neuropathy noted in CD is chronic, symmetric distal neuropathy, but autonomic neuropathy, chronic inflammatory demyelinating neuropathy, acute inflammatory demyelinating neuropathy (Guillain-Barre syndrome), and mononeuritis multiplex have also been described [36]. Rates of peripheral neuropathy in pediatric CD range form 0.1–7.4% [37,38,39]. It is speculated there may be an autoimmune cause for the neuropathy as many studies have located anti-ganglioside antibodies in these patients [40]. It is also possible that the nutritional deficiencies common to CD patients may account for the neuropathy. There has been great discrepancy as to the effectiveness of the GFD on resolution of peripheral neuropathies in celiac disease, ranging from findings of great symptom improvement [41], to only subjective improvements [42], to little to no improvements at all [43]. Intravenous immunoglobulin, plasmapheresis and etanercept do not appear to be effective in celiac related peripheral neuropathies [42]. 

While cerebellar ataxia is a well-recognized extra-intestinal manifestation of CD in adults occurring in as many as 40% of adult CD patients, it is far less common in children with rates that are closer to 0.068–1.79% [44,45,46]. The clinical presentation is indistinguishable from other forms of cerebellar ataxia with progressive unsteadiness of the gait and limbs. Both sexes appear to be affected equally, the mean age of onset is 53 years, and cerebellar atrophy can be detected by brain magnetic resonance imaging (MRI) in a vast number of adult patients as well [47]. On the contrary cerebellar atrophy appears to be exceptionally rare in children. While children are more prone to developing unilateral or bilateral focal hyper-intense white matter lesions, actual cerebellar atrophy is very uncommon [45,46]. The severity of cerebellar atrophy appears to correlate with the duration of exposure to gluten likely explaining the decreased rates and severity of cerebellar atrophy seen in children [48]. Similar to other neurologic manifestations in celiac patients, the effects of the GFD on recovery are highly variable [49], and if there have been no improvements on the diet within a year or the ataxia is rapidly progressive, the use of intravenous immunoglobulins has been reported to provide possible benefits [50]. 

Most pediatric studies have failed to find and increase the prevalence of epilepsy in CD as compared with the general population [37]. Epilepsy specifically associated with cerebral calcifications, though, has been associated with CD. It was first reported in the pediatric literature in 1994 by Pascotto, et al. who described four separate patients with epilepsy with cerebral calcifications refractory to medical management, followed between 1980 and 1990 at the Clinic of Child Neuropsychiatry of Naples University. Despite extensive testing, an origin for the calcifications was not discovered. These patients later underwent celiac testing, 1 for failure to thrive (FTT), while the other three had no obvious gastrointestinal complaints. Two of the four patients, including the patient with FTT, had elevated titers of immunoglobulin A (IgA) and Immunoglobulin G (IgG) antigliadin antibodies and also demonstrated histologic findings of crypt hyperplasia, alterations of superficial epithelium with picnotic, cubic cells, and remarkable lymphoplasmacellular infiltration, as is consistent with the diagnosis of celiac disease. Following diagnosis, these two patients started a strict GFD with reverse of the FTT and initial improvements in the seizure frequency, but worsened again roughly six months later [51]. A subsequent, similar case report was published in 2012. In this case, the child was positive for endomysial (EMA) immunoglobulin IgA, gliadin (DGP) IgA, and IgG and transglutaminase (tTG) two IgA and IgG antibodies. Endoscopic biopsies showed subtotal villous atrophy in the duodenum confirming the diagnosis. Additionally, high levels of antibodies to tTG six IgA and EMA (IgA) were also found in the cerebrospinal fluid. The child was placed on a GFD and was seizure free for 18 months at the time of the publication of the paper [52]. 

## 10. Behavioral Changes and Psychiatric Disorders

A wide range of psychiatric symptoms and disorders have been associated with CD, including anxiety disorders, depressive disorders, attention deficit hyperactivity disorders (ADHD), and autism spectrum disorders (ASD). While psychiatric disorders that occur after the diagnosis of celiac disease has been made are more often associated with an impaired quality of life and difficulty adapting to the chronic nature of the disease [53], psychiatric disorders that occur prior to the diagnosis of celiac disease have been hypothesized to be related to disease related cerebral hypoperfusion [54], proinflammatory cytokines [55], and low folate levels [56]. We will focus on anxiety and depressive disorders within this review as to date, no greater rates of ADHD and ASD have been found in the celiac population as compared to the general population [57,58,59]. 

Rates of psychiatric disorders, including anxiety and depression, in pediatric celiac patients range from 5–10% [1,60], substantially lower than those found in adults [61]. A recent study by Smith et al. in 2018 examined maternal reports of their children’s psychological functioning over 3.5 years in celiac children aged 2–3 years who were persistently positive for tTG IgA as compared to children with normalized tTG IgA. These serology results were obtained in a retrospective manner by sampling of stored blood. The mothers were blinded to the results of the serological testing. Mothers completed the Achenbach Child Behavior Checklist, a well-validate questionnaire to measure behavioral and emotional function in preschoolers aged 1.5–5 years old. There was a statistically significant difference in the scores from mothers of celiac children with persistently elevated tTG IgA as compared to those with normalized tTG IgA. Celiac children with persistently elevated tTG IgA scored significantly higher for anxiety, depression, aggressive behaviors, and sleep problems. Initiation of the GFD did not appear to have an impact on the psychological functioning of the patients in this study (though very young children) [62]. Another study by Simsek et al. in 2015 assessed the rates of psychiatric symptoms in newly diagnosed celiac pediatric patients aged 9 to 16 years old as compared to age and sex matched healthy controls that had presented for routine checkup. On the contrary to the previous study, this study showed no statistically significant differences between depression scores in newly diagnosed CD patients and controls, though there were statistically lower scores on emotional well within the CD patients as compared to the controls. Patients on a strict GFD showed significant reductions in follow up depression scores as compared to patients that were non-compliant with the diet [63]. There have been very mixed results, though, on improvements in depression once on a GFD ranging from no change in depression [53], to moderate improvements in depression [1], to the complete resolution of depression [64]. 

## 11. Alopecia

While alopecia has an association with pediatric CD, it is one of the less common extra-intestinal manifestations seen, occurring in roughly 1% of patients [1]. It is presumed to occur through an autoimmune reaction involving T-cell dysregulation and can lead to patchy loss of skull hair (alopecia areata), total loss of skull and facial hair (alopecia totalis), and total loss of full body hair (alopecia universalis). It is speculated that there are autoantibodies directed against anagen-stage hair follicle structures and a direct association with the human leukocyte antigen (HLA)-DQB1*0201 allele. A number of studies have assessed the involvement of alopecia areata in CD patients. Rates of alopecia areata appear to be equally distributed between male and female celiac patients, roughly 40% have a coinciding alternate autoimmune disorder, 70% had gastrointestinal symptoms in addition to the alopecia, and 100% of patients had positive celiac serologies (tTG IgA or EMA IgA) with subtotal or total villous atrophy on duodenal biopsies. Trials of oral zinc and other topical treatments were attempted with minimal response. Of the patients who started a strict GFD, 26% had no regrowth, 22% had a partial regrowth, and 52% had the complete regrowth of hair by 12–24 months (most within the first 2–3 months). All of the patients had a normalization of their celiac serologies and duodenal biopsies by 24 months, despite the growth or lack of growth of hair. Younger patients and shorter lag times between the onset of alopecia areata and initiation of the GFD led to a more favorable hair growth response. Sex, lack of GI symptoms, and the severity of biopsy results did not appear to impact whether a patient would or would not have regrowth of hair [65,66,67,68,69,70,71].

## 12. Treatment of the Extra-Intestinal Manifestations of CD

Strict, lifelong adherence to a GFD remains the only available treatment for patients that are diagnosed with CD, and, as stated previously, should result in a complete return to health in the majority of patients, especially pediatric. Other pharmacological therapies are being evaluated for the treatment of CD, including enzymes, to inactivate immunogenic gluten peptides in the human gastrointestinal tract [72], agents that sequester gluten in the lumen [73], modulators of gut permeability [74], and of antigen presentation and immune responses, including those that block tTG [75] and HLA [76], IL-15 inhibitors [77], and the development of vaccines that are able to induce oral tolerance to gluten [78]. Of these treatment options, the only one that is currently on the market is the gluten-specific enzyme, GliadinX (AN-PEP). Unfortunately, it is only capable of detoxifying 0.2 g of gluten or roughly that of 1/8 of a slice of gluten-containing bread. For this reason, it should only be used as an adjunct to the GFD when there are concerns for accidental gluten contamination and in an effort to ameliorate symptoms, not as a replacement for the GFD.

While these pharmacological options are promising, it is still unclear how well they will work to minimize gut inflammation and alleviate gastrointestinal, and, the even more complex extra-intestinal manifestations of CD. While it is likely that some of the alternatives may come to fruition in the next year or two, a true “cure”, although certainly possible, might take much longer [79].
